# Dietary antioxidants and obesity: a new perspective on the role of composite dietary antioxidant index in reducing obesity risk using a dual-criteria definition

**DOI:** 10.3389/fnut.2025.1600925

**Published:** 2025-07-31

**Authors:** Yi-Qian Chen, Chen Wang, Yi-Jie Zhang, Yue-Yang Zhang

**Affiliations:** Department of Nursing, Beijing Health Vocational College, Beijing, China

**Keywords:** composite dietary antioxidant index, obesity, NHANES, BMI, waist circumference

## Abstract

**Objective:**

To investigate the association between the Composite Dietary Antioxidant Index (CDAI) and obesity defined by both BMI and waist circumference (WC).

**Methods:**

This was a cross-sectional study using data from the National Health and Nutrition Examination Survey (NHANES) 2009–2018. Multivariable logistic regression models and restricted cubic splines were used to assess the relationship between CDAI and obesity, defined as BMI ≥ 30 kg/m^2^ combined with WC ≥ 88 cm (women) or ≥102 cm (men). Models were adjusted for sociodemographic, lifestyle, and comorbidity factors. A stratified analysis and sensitivity analysis were also conducted.

**Results:**

Among 17,067 participants, CDAI was inversely associated with obesity (OR: 0.97, 95% CI: 0.95–0.99). Participants in the highest CDAI quartile had a 23% lower obesity risk compared to the lowest quartile (OR: 0.77, 95% CI: 0.62–0.95). Subgroup and sensitivity analyses yielded consistent results.

**Conclusion:**

CDAI is inversely associated with obesity defined by dual criteria, suggesting that dietary antioxidants may play a protective role in obesity prevention. By using a more comprehensive definition of obesity, our study provides insights that are more directly applicable to clinical practice and public health strategies aimed at reducing obesity-related morbidity and mortality.

## 1 Introduction

Obesity is a multifaceted global health crisis that has reached epidemic proportions, affecting nearly every aspect of public health (1). Traditional definitions of obesity, primarily based on Body Mass Index (BMI), have long been the cornerstone for identifying and managing this condition (2). However, using BMI alone as a diagnostic criterion for obesity has certain limitations (3). The latest consensus, which divides obesity into preclinical obesity, obesity, and clinical obesity, emphasizes that the assessment of obesity should be based on BMI and should be combined with at least one other anthropometric indicator, such as waist circumference (WC) (4). WC provides critical complementary information by directly quantifying abdominal fat deposition, which is independently associated with insulin resistance, dyslipidemia, and cardiovascular mortality (5). Venkatrao et al. combined BMI and WC to provide a more accurate assessment of obesity status (5). This new definition not only accounts for overall adiposity but also considers the distribution of body fat, which is crucial for understanding the associated health risks, particularly those related to abdominal obesity.

In parallel with the evolving definition of obesity, there has been growing interest in the role of oxidative stress in the pathogenesis of obesity and its related comorbidities. Oxidative stress, resulting from an imbalance between the production of reactive oxygen species and the body’s antioxidant defenses, has been implicated in the development of obesity and metabolic dysfunction (6, 7). Elevated levels of oxidative stress can lead to inflammation, insulin resistance, and adipogenesis, which are key factors in the pathogenesis of obesity (8–10). The Composite Dietary Antioxidant Index (CDAI) is a comprehensive measure that evaluates the overall antioxidant characteristics of an individual’s diet by summarizing the intake of these key antioxidants (11). Previous studies have demonstrated that higher CDAI scores are associated with a reduced risk of several chronic diseases, including hypertension (12), stroke (13), cardiovascular disease (14, 15), depression (16), and cancer (17). Although there are also some researches on CDAI and obesity (18–20), the relationship between CDAI and obesity, particularly when defined by the latest clinical criteria, remains underexplored.

Given the established link between oxidative stress and obesity, and the potential protective role of dietary antioxidants, we hypothesize that a higher CDAI score is associated with a lower prevalence of obesity when defined by the latest clinical criteria integrating BMI and WC. This study aims to investigate the association between CDAI and obesity in a nationally representative sample of US adults using data from the National Health and Nutrition Examination Survey (NHANES). By exploring this relationship using the updated definition of obesity, we hope to provide novel insights into the potential role of dietary antioxidants in obesity prevention and management.

## 2 Methods

### 2.1 Data source and study population

This analysis utilized data from the NHANES conducted between 2009 and 2018. NHANES, a population-based surveillance system conducted by the National Center for Health Statistics. The study design incorporates stratified, multistage probability cluster sampling methodology to evaluate health and nutritional parameters among community-dwelling U. S. adults (21). Standardized instruments captured socio-demographic profiles, health behaviors, and medical histories through structured interviews conducted during enrollment (22). The questionnaires were administered and collected at study recruitment by trained interviewers. Certified staff implemented data collection protocols at baseline assessments. Biological specimens and anthropometric evaluations were performed by licensed healthcare personnel within dedicated mobile research units. Deidentified NHANES datasets are publicly accessible through its official portal.^1^ Ethical compliance was ensured through review by the National Center for Health Statistics’ Institutional Review Board (IRB), with written informed consent obtained from all enrollees. Secondary utilization of existing data qualified for IRB exemption (23). Individuals over 20 years old who had completed an interview participated in our study. Eligibility criteria required participants to be aged ≥20 years with completed baseline evaluations. Exclusion criteria comprised pregnancy status or incomplete records regarding the CDAI, BMI, WC, or covariates. Methodological reporting adhered to STROBE guidelines for observational cross-sectional research.

### 2.2 Exposure and outcome

#### 2.2.1 Composite dietary antioxidant index

The NHANES collected participants’ food intake data through two non-consecutive 24-h dietary recall. Initial face-to-face interviews occurred at the Mobile Examination Centers (MEC), with telephone follow-ups conducted 3–10 days later. Mean daily consumption values were calculated by averaging both dietary records. CDAI scores were generated following Wright et al.’s modified methodology (11). This composite metric integrates standardized daily intakes of zinc, selenium, carotenoids, vitamin A, vitamin C, vitamin E. Each micronutrient’s intake was standardized by subtracting population means and dividing by standard deviations.

#### 2.2.2 BMI-WC-obesity

According to WHO standards, BMI ≥ 30 kg/m^2^ was defined as obese; WC ≥ 88 cm (female) or ≥102 cm (male) was defined as abdominal obesity; and Waist-Height Ratio (WHtR) ≥ 0.5 was defined as obese. According to the latest definition and diagnosis of clinical obesity (4), the assessment of obesity needs to include at least one other anthropometric indicator in addition to BMI, the BMI-WC-Obesity in this study was defined as BMI ≥ 30 kg/m^2^ and, female WC ≥ 88 cm or male WC ≥ 102 cm.

### 2.3 Covariates

Based on previous studies and clinical experience, potential variables that may confound the relationship between CDAI and obesity were included as covariates in this study, including demographic factors and health status factors. Demographic factors included age, gender, race, educational level, marital status, and family income. Health status factors included lifestyles (smoking, drinking, physical activity, and daily energy intake) and comorbidities (hypertension, hypercholesterolemia, diabetes, cardiovascular disease, and stroke). Detailed protocols are available at https://www.cdc.gov/nchs/ nhanes, which was accessed on August 24, 2024. A description of these covariates is provided in Supplementary Table S1.

### 2.4 Statistical analysis

Accounting for NHANES’s multistage probability sampling framework, weighted analytical approaches were systematically applied per recommended protocols (24). In this study, weight computation followed the equation: Dietary Day 2 weights × 1/5 across 2009–2018 survey cycles.

Categorical variables were expressed as proportions (%) while continuous variables used mean±SD or median (IQR). Comparative group analyses employed ANOVA for normal distributed data, Kruskal-Wallis tests or skewed distributions, and Pearson *χ*^2^ tests for categorical comparisons. Furthermore, UpSet visualizations delineated disease-state interrelationships. With minimal missingness (≤8.6% across covariates), imputation was deemed unnecessary.

Multivariable logistic regression analyses estimated odds ratios (OR) with 95 percent confidence intervals (95% CIs) for the relationship between CDAI and BMI-WC-Obesity with adjustment for potential confounding variables. Multicollinearity was tested using the variance inflation factor (VIF) method, with a VIF of 5 or more indicating the presence of multicollinearity. In the crude model, we did not adjust any covariates. Model 1 adjusted sociodemographic variables (age, sex, race, educational level, family income, and marital status) and NHANES cycles. Model 2 adjusted sociodemographic variables, NHANES cycles, and lifestyle variables (smoking status, drinking status, physical activity, and calorie consumption). Model 3 was fully adjusted, including sociodemographic variables, NHANES cycles, lifestyle habits, and comorbidities (hypertension, hypercholesterolemia, diabetes, CVD, and stroke). In addition, restricted cubic spline (RCS) were used to assess potential non-linear associations between CDAI and obesity outcomes. Four knots were placed at the 5th, 35th, 65th, and 95th percentiles of CDAI distribution. This knot configuration was chosen to reflect the data distribution, balance model flexibility with interpretability, and is consistent with prior epidemiologic studies employing spline-based modeling.

To assess the stability of our findings, stratified analyses such as subgroup and interaction analyses were performed according to age, gender, race, education level, marital status, and family income. Furthermore, several sensitivity analyses were performed. First, CDAI were analyzed as continuous variates and also, respectively, divided into quartiles. Second, we conducted a sensitivity analysis based on the different definations of obesity. Third, we conducted a sensitivity analysis that excluded participants with extreme CDAI intake, which winsorized values above the 1st percentile to the 99th percentile. Fourth, we conducted sensitivity analyses for each CDAI component (vitamins A, C, E, zinc, selenium, carotenoids) using multivariable logistic regression models identical to those applied for the composite CDAI.

Because the sample size was determined solely on the data provided, no *a priori* statistical power estimates were performed. All statistical analyses were performed with R, version 4.2.2 (R Project for Statistical Computing) and with Free Software Foundation statistics software, version 2.1. In all tests, *p* < 0.05 (2-sided) was considered to indicate statistical significance.

## 3 Results

### 3.1 Characteristics of the participants

**Figure 1:**
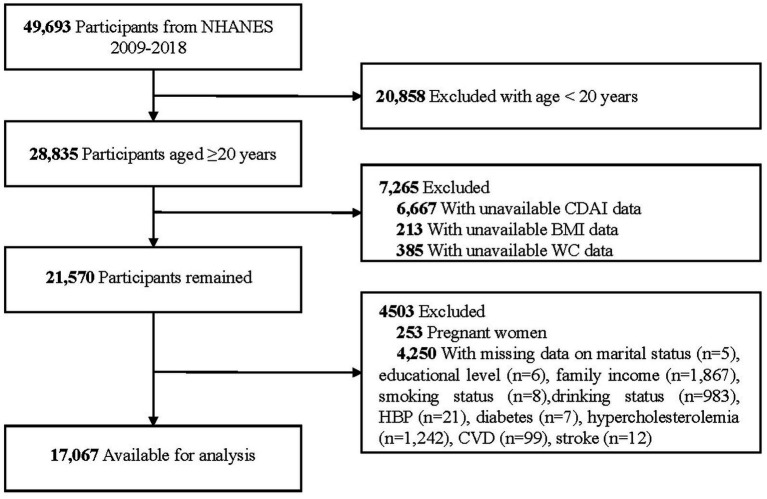
Flow chart of sample selection from the NHANES 2009–2018. NHANES, National Health and Nutrition Examination Survey; CDAI, Composite Dietary Antioxidant Index; BMI, Body Mass Index; WC, Waist Circumference; CVD, Cardiovascular Disease.

Among the 28,835 participants aged ≥20 years, 7,265 were excluded due to missing or implausible data related to CDAI, BMI, and WC. Additionally, 253 pregnant women and 4,250 individuals with missing or invalid covariate data were excluded, resulting in a final analytic sample of 17,067 participants. The detailed inclusion and exclusion criteria are depicted in Figure 1.

Supplementary Table S2 presents the baseline characteristics of all subjects stratified by CDAI intake quartiles. BMI-WC-Obesity was present in 7,002 participants (39.9%). Weighted analyses revealed that the mean age of the 17,067 participants was 48.3 years (SD, 16.7), and 8,757 (50.8%) were female. Individuals with higher CDAI intake were more likely to be male, non-Hispanic White, have a higher educational level, be married or living with a partner, have a higher family income, be nonsmokers, be drinkers, engage in less vigorous activity, consume more calories, have a lower prevalence BMI-WC-Obesity, and exhibit lower values for BMI, WC, and WHtR.

**Figure 2:**
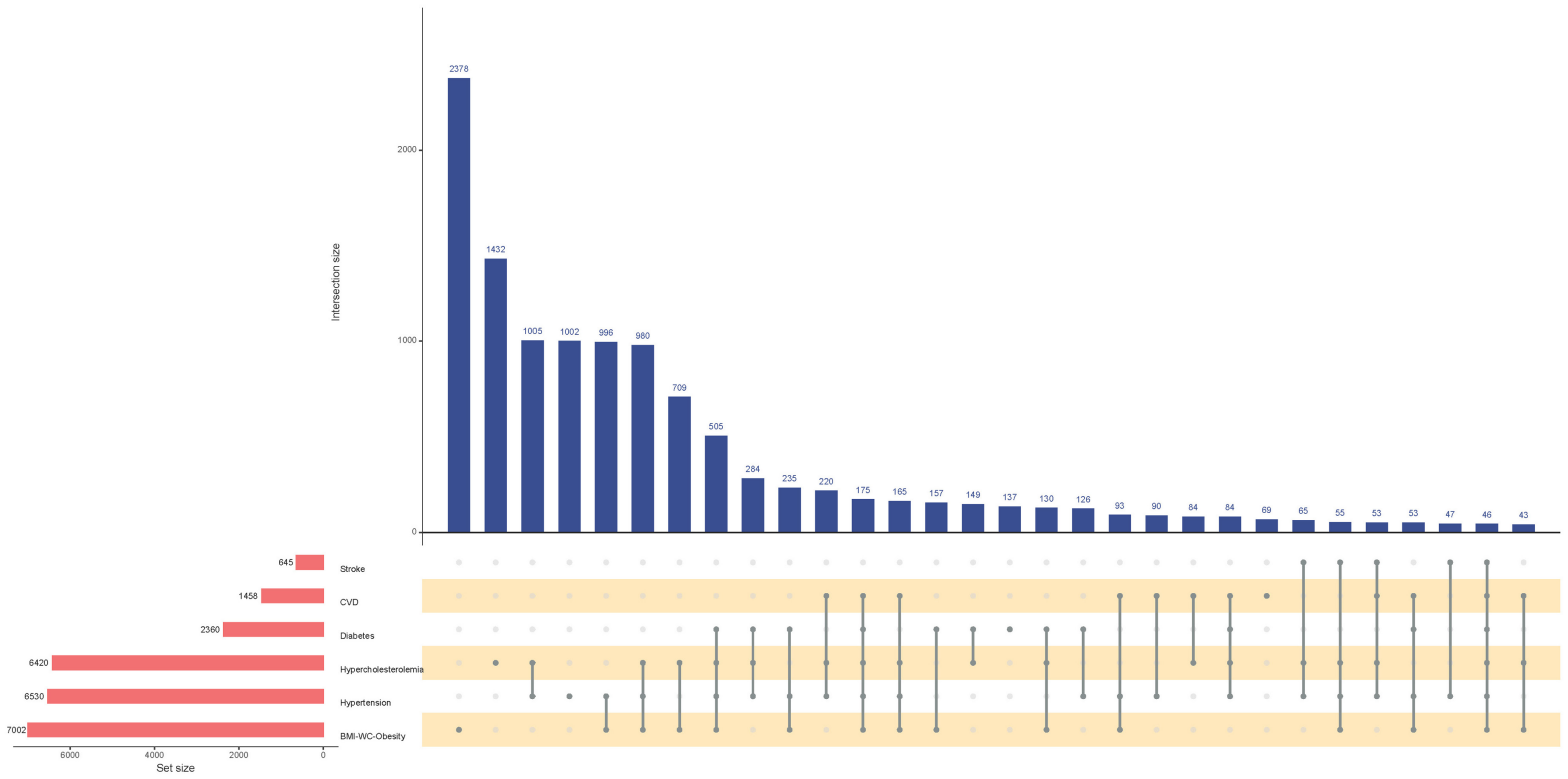
UpSet Plot of the Comorbidity Patterns of Stroke, Cardiovascular Disease, Diabetes, Hypercholesterolemia, Hypertension, and BMI-WC-Based Obesity. CVD, Cardiovascular Disease; BMI, Body Mass Index; WC, Waist Circumference. Horizontal bar on left represents number of set size in each covariates. Dots and lines represent subsets of set size. Vertical histogram represents number of set size in each subset.

The UpSet plot (Figure 2) effectively visualizes complex cardiometabolic comorbidity patterns in the obese population. The left-side bar chart illustrates the total number of individuals affected by each specific condition. Bar heights in the main matrix quantify the prevalence of specific disease combinations. Notably, analysis revealed that only 2,378 (34.0%) obese individuals had obesity without any specified comorbidities, while 505 (7.2%) presented with the specific comorbidity combination of concurrent hypertension, hypercholesterolemia, and diabetes.

### 3.2 Multivariable regression analyses

**Figure 3:**
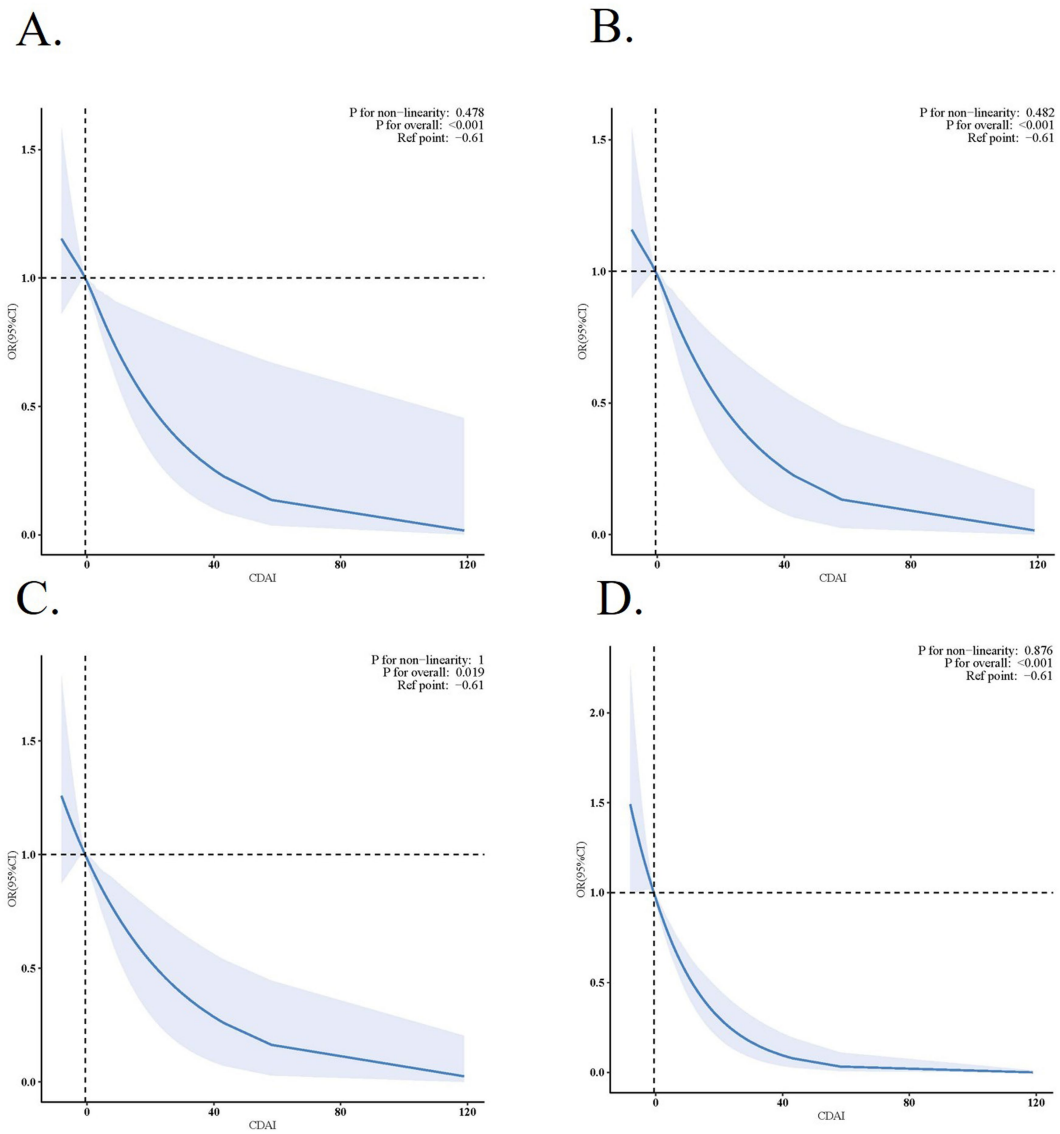
Association between CDAI and Obesity. **(A)** Association between CDAI and BMI-WC-Obesity. **(B)** Association between CDAI and BMI defined obesity. **(C)** Association between CDAI and WC defined obesity. **(D)** Association between CDAI and WHtR defined obesity. Solid and dashed lines represent the predicted value and 95% confidence intervals. They were adjusted for age, sex, race and ethnicity, educational level, family income, marital status, NHANES cycles, smoking status, drinking status, physical activity, calorie consumption, hypertension, highcholesterol, diabetes, cardiovascular disease and stroke.

The results from sample-weighted multivariable regression analyses are presented in Table 1. VIF for all included variables were below 5, demonstrating no significant multicollinearity concerns (Supplementary Table S2). CDAI, as a continuous variable, was inversely associated with BMI-WC-Obesity in all 3 models [OR (95% CI), 0.96 (0.95, 0.97); 0.97 (0.96, 0.99); 0.97 (0.95 to 0.99)]. When compared with the Q1, the Q4 of CDAI was inversely associated with BMI-WC-Obesity in all 3 models [OR (95% CI), 0.77 (0.66, 0.90); 0.79 (0.65, 0.97); 0.77 (0.62, 0.95)]. Accordingly, restricted cubic spline (RCS) regression was performed with 4 knots at the 5th, 35th, 65th, and 95th percentiles of CDAI to assess linearity and examine the dose–response curve between CDAI and BMI-WC-Obesity after adjusting variables in Model 3 (Figure 3A).

**Table 1:** Association of CDAI with BMI-WC-obesity among participants in the NHANES 2009–2018 cycles.

CDAI (Categories /Continuous)	Crude		Model 1^a^		Model 2^b^		Model 3^c^	
	OR (95% CI)	*p* value	OR (95% CI)	*p* value	OR (95% CI)	*p* value	OR (95% CI)	*p* value
Q1	Ref	–	Ref	–	Ref	–	Ref	–
Q2	0.91 (0.83, 0.99)	0.026	0.95 (0.83, 1.10)	0.506	0.96 (0.82, 1.11)	0.563	0.95 (0.81, 1.11)	0.498
Q3	0.84 (0.77, 0.92)	<0.001	0.94 (0.83, 1.06)	0.307	0.94 (0.82, 1.08)	0.405	0.93 (0.81, 1.08)	0.337
Q4	0.68 (0.62, 0.74)	<0.001	0.77 (0.66, 0.90)	<0.001	0.79 (0.65, 0.97)	0.022	0.77 (0.62, 0.95)	0.017
CDAI	0.96 (0.95, 0.97)	<0.001	0.97 (0.96, 0.99)	<0.001	0.97 (0.96, 0.99)	0.003	0.97 (0.95, 0.99)	<0.001
Trend test	–	<0.001	–	0.002	–	0.027	–	0.017

NHANES, National Health and Nutrition Examination Survey; CDAI, Composite Dietary Antioxidant Index; BMI, Body Mass Index (calculated as weight in kilograms divided by height in meters squared); WC, Waist Circumference; OR, Odds ratio; CI, Confidence interval; Q, Quartiles. ^a^Adjusted for sociodemographic variables (age, sex, race and ethnicity, educational level, family income, and marital status) and NHANES cycles. ^b^Adjusted for sociodemographic variables, NHANES cycles, and lifestyle variables (smoking status, drinking status, physical activity, and calorie consumption). ^c^Adjusted for sociodemographic variables, NHANES cycles, lifestyle habits, and comorbidities (hypertension, hypercholesterolemia, diabetes, cardiovascular disease and stroke).

### 3.3 Subgroup analyses

**Figure 4:**
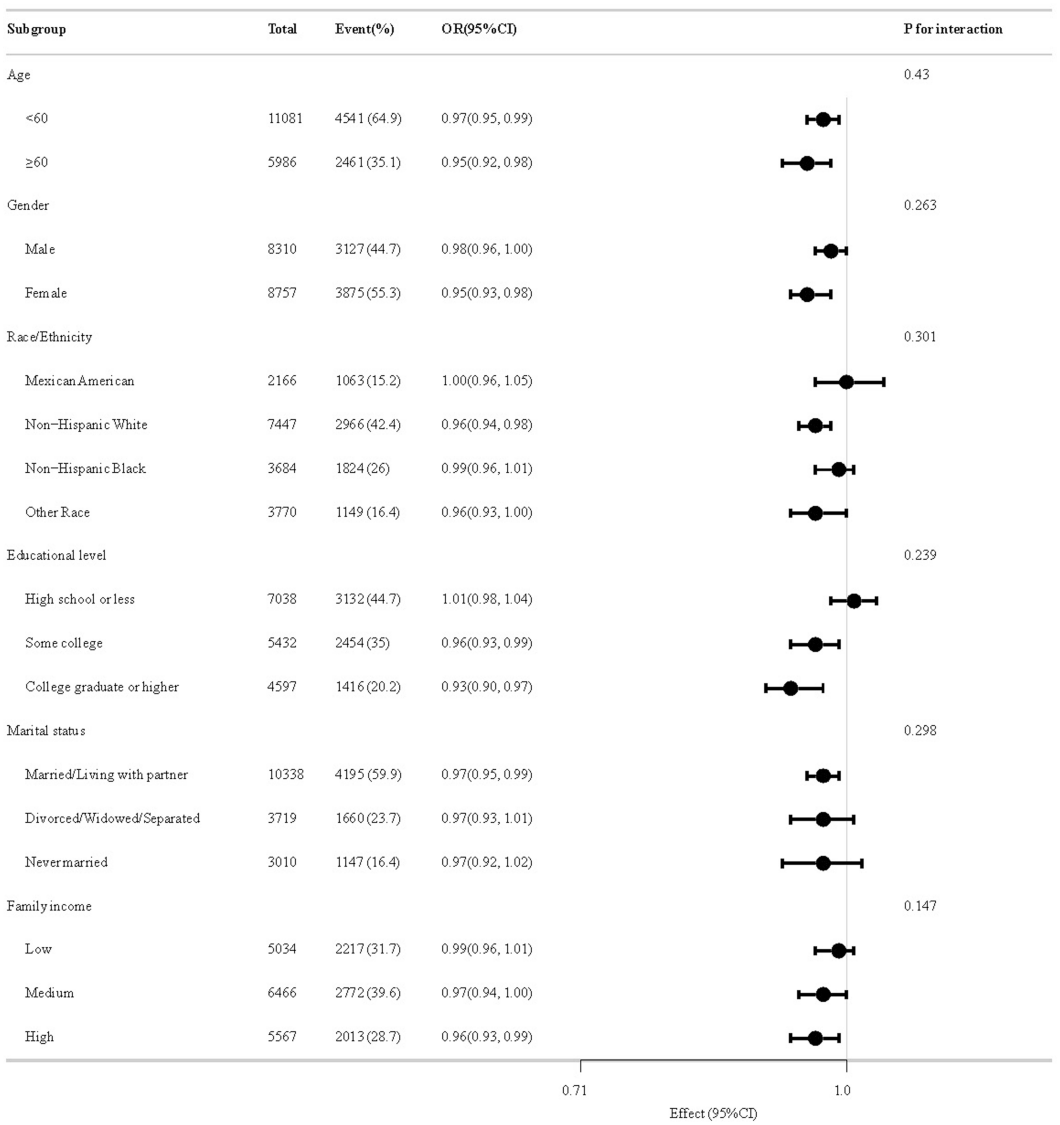
The relationship between CDAI and BMI-WC-Obesity according to basic features.

Figure 4 delineates subgroup analytical outcomes. In several subgroups, stratified analysis was performed to assess potential effect modifications on the relationship between CDAI and BMI-WC-Obesity. Across all population strata, no statistically meaningful interaction effects emerged post-stratification (*p* > 0.05).

### 3.4 Sensitivity analyses

The results of sensitivity analyses are summarized in Supplementary Table S3; Figures 3B–D. When obesity was defined by BMI, WC, and WHtR respectively, after adjusting for sociodemographic characteristics, NHANES cycles, lifestyle variables, and comorbidities, the association between CDAI and obesity remained [OR (95% CI), 0.97 (0.95, 0.99); 0.97 (0.95, 0.99); 0.94 (0.92, 0.96)]. In addition, after excluding participants with extreme CDAI intake, the adjusted OR of BMI-WC-Obesity was 0.97 (95% CI, 0.95–1.00; *p* = 0.023). Association of six components of CDAI and BMI-WC-Obesity are presented in Table 2. Vitamins A, C, E, and carotenoids showed significant inverse associations with BMI-WC-obesity in fully adjusted models [OR (95% CI), 0.93 (0.9, 0.97); 0.94 (0.91, 0.98); 0.96 (0.92, 0.99); 0.95 (0.91, 0.98)].

**Table 2:** Association of six components of CDAI and BMI-WC-obesity.

Components	Crude		Model 1^a^		Model 2^b^		Model 3^c^	
	OR (95% CI)	*p* value	OR (95% CI)	*p* value	OR (95% CI)	*p* value	OR (95% CI)	*p* value
Vitamins A	0.88 (0.85, 0.92)	<0.001	0.92 (0.89, 0.96)	<0.001	0.94 (0.90, 0.97)	<0.001	0.93 (0.9, 0.97)	<0.001
Vitamins C	0.89 (0.86, 0.92)	<0.001	0.92 (0.89, 0.95)	<0.001	0.93 (0.90, 0.97)	<0.001	0.94 (0.91, 0.98)	0.001
Vitamins E	0.90 (0.87, 0.93)	<0.001	0.94 (0.91, 0.97)	<0.001	0.96 (0.93, 1.00)	0.042	0.96 (0.92, 0.99)	0.019
Zinc	0.92 (0.89, 0.95)	<0.001	0.98 (0.95, 1.01)	0.245	1.00 (0.96, 1.04)	0.896	1.00 (0.96, 1.04)	0.903
Selenium	0.94 (0.92, 0.97)	<0.001	1.01 (0.98, 1.05)	0.438	1.08 (1.03, 1.13)	0.002	1.04 (0.99, 1.09)	0.091
Carotenoids	0.89 (0.86, 0.92)	<0.001	0.94 (0.91, 0.97)	<0.001	0.95 (0.92, 0.99)	0.005	0.95 (0.91, 0.98)	0.002

OR, Odds ratio; CI, Confidence interval; CDAI, Composite Dietary Antioxidant Index; BMI, Body Mass Index; WC, Waist Circumference. ^a^Adjusted for sociodemographic variables (age, sex, race and ethnicity, educational level, family income, and marital status) and NHANES cycles. ^b^Adjusted for sociodemographic variables, NHANES cycles, and lifestyle variables (smoking status, drinking status, physical activity, and calorie consumption). ^c^Adjusted for sociodemographic variables, NHANES cycles, lifestyle variables, and comorbidities (hypertension, hypercholesterolemia, diabetes, cardiovascular disease and stroke).

## 4 Discussion

This study investigated the relationship between CDAI and obesity, employing a comprehensive definition that integrates BMI and WC in a large, nationally representative cohort of U. S. adults. Our analysis revealed a significant inverse association between CDAI and BMI-WC-Obesity, which remained consistent across multiple statistical models, subgroup analyses, and sensitivity analyses. These findings suggest that a higher intake of dietary antioxidants, as quantified by CDAI, may play a protective role in mitigating obesity risk.

While previous research has explored the associations between dietary antioxidants and various obesity-related indicators (18–20, 25), the specific relationship between CDAI and BMI-WC-Obesity (4), has not been thoroughly examined. Our study addresses this gap by demonstrating that higher CDAI levels are associated with a lower prevalence of BMI-WC-Obesity, consistent with prior research (19). This association is biologically plausible. Experimental evidence suggests that antioxidants may attenuate adipogenesis by modulating key signaling pathways such as AMPK and PPARγ, improve insulin sensitivity via reducing inflammatory cytokines (e.g., TNF-*α*, IL-6), and enhance mitochondrial biogenesis through PGC-1α activation. These mechanisms collectively mitigate oxidative stress-induced metabolic dysfunction (26–28). Clinically, the UpSet plot helped stratify obesity-related risk phenotypes, highlighting that individuals with comorbid hypertension, diabetes, and hypercholesterolemia may experience amplified oxidative stress. For such populations, dietary antioxidant interventions may provide dual benefits by both alleviating central obesity and reducing cardiometabolic burden.

A key strength of our study lies in the adoption of a dual-criteria definition of obesity, incorporating both BMI and WC, in alignment with the latest recommendations from The Lancet Diabetes & Endocrinology Commission (4). This approach addresses the limitations of using BMI alone, such as its inability to distinguish between fat and muscle mass or to account for visceral fat (29, 30). By utilizing this more nuanced definition, our study enhances the clinical relevance of its findings, more accurately capturing central obesity and its associated cardiometabolic risks, and reducing the misclassification of individuals with normal BMI but elevated visceral fat. In addition, we included WHtR as an alternative measure of central adiposity. WHtR is a simple and widely used index that relates waist circumference to height, offering an efficient proxy for fat distribution. To further validate the robustness of our findings, we conducted sensitivity analyses using alternative obesity definitions, including BMI, WC, and WHtR.

Our component-specific analysis suggests that vitamins A, C, E and carotenoids contribute dominantly to CDAI’s protective effect against obesity. This supports prioritizing foods rich in these antioxidants—such as citrus fruits, carrots, and nuts. Notably, as no individual component reproduced the full effect size of the composite CDAI, we advocate for a whole-diet approach. Clinicians could use CDAI thresholds to identify suboptimal antioxidant intake in high-risk patients and tailor nutrition counseling accordingly.

Subgroup analyses did not identify significant interactions based on age, sex, race, education level, marital status, or family income. This consistency across diverse demographic and lifestyle factors underscores the generalizability of the inverse association between CDAI and BMI-WC-Obesity. Although no statistically significant effect modification was observed, the direction and magnitude of association in certain subgroups—particularly those with lower antioxidant exposure—suggests that these populations might derive greater benefit from dietary interventions. Given the well-documented disparities in obesity prevalence across racial/ethnic groups and the influence of socioeconomic status on dietary patterns (31–33), this finding is particularly significant. Nonetheless, future research should continue to explore potential effect modifiers in larger and more diverse populations to validate these results further.

In addition to subgroup effects, we also examined the dose–response pattern between CDAI and obesity risk. Our restricted cubic spline analysis confirmed a linear inverse dose–response relationship between CDAI and BMI-WC-Obesity, implying that even modest increases in antioxidant intake may be protective. The linear inverse association suggests that even modest increases in dietary antioxidant intake may confer protective effects against obesity. This insight is especially pertinent for public health initiatives aimed at promoting diets rich in fruits, vegetables, and whole grains, which are abundant sources of antioxidants (34).

Despite its strengths, this study is not without limitations. First, the cross-sectional design precludes causal inferences. Although our findings suggest a protective role of dietary antioxidants against obesity, longitudinal or interventional studies are necessary to establish causality. Second, the sample size was determined by available NHANES participants, and no *a priori* power analysis was conducted. Nonetheless, we acknowledge that *post hoc* power estimation may offer some interpretive value, particularly in understanding nonsignificant findings in subgroup analyses. Third, the reliance on self-reported dietary data through 24-h recalls may introduce substantial recall bias. Although NHANES mitigates these issues via two non-consecutive recalls, certified interviewer training, and visual portion aids, residual reporting inaccuracies remain unavoidable. We also now suggest using biomarkers or validated Food Frequency Questionnaire (FFQs) in future studies. Fourth, residual confounding from unmeasured factors, such as genetic predisposition or environmental exposures, cannot be entirely ruled out. Future research integrating genetic and environmental data may provide further clarity on these interactions. Fifth, although CDAI provides a useful measure of dietary antioxidant intake, the absence of oxidative stress biomarkers (e.g., F2-isoprostanes) in the NHANES dataset limits our ability to directly link dietary patterns to underlying biological mechanisms. Future studies should incorporate such biomarkers and consider multi-omics approaches to gain a more comprehensive understanding of the antioxidant-obesity relationship.

Given the linear inverse CDAI-obesity association, we advocate Mediterranean-style diets emphasizing vitamin C (citrus/berries), vitamin E (nuts/oils), carotenoids (carrots/spinach), and zinc/selenium (whole grains/seafood). Public health strategies could incorporate CDAI into community nutrition screening, align it with food policies (e.g., fresh produce subsidies), and deploy digital tools to guide antioxidant intake optimization in high-risk groups. Beyond immediate applications, future research could also explore methodological innovations. For instance, machine learning approaches may be used to integrate diverse anthropometric and imaging-derived indicators (e.g., BMI, WHtR, and visceral fat area) to develop more advanced obesity phenotypes. Such multidimensional profiling could help identify subtypes with differential responses to dietary antioxidant interventions, ultimately enabling precision nutrition strategies tailored to individual risk profiles.

## 5 Conclusion

In summary, our study provides robust evidence of an inverse association between CDAI and BMI-WC-Obesity, highlighting the potential protective role of dietary antioxidants in obesity prevention. By employing a multidimensional definition of obesity, advanced statistical techniques, and rigorous sensitivity analyses, our findings contribute valuable insights to the literature on the role of diet in metabolic health. Future research should focus on elucidating causal mechanisms and translating these findings into effective public health interventions.

## Data Availability

Publicly available datasets were analyzed in this study. This data can be found at: https://www.cdc.gov/nchs/nhanes/index.htm.

## Footnotes

^1^http://www.cdc.gov/nchs/nhanes/index.htm, accessed on 24 August 2024.
