# Supplementary material for: Dietary antioxidants and obesity: a new perspective on the role of composite dietary antioxidant index in reducing obesity risk using a dual-criteria definition
Source: Front Nutr. 2025 Jul 31;12:1600925. doi: 10.3389/fnut.2025.1600925 (PMC12350136; doi:10.3389/fnut.2025.1600925)
Supplement: Supplementary file 1 [file Table_1.docx]

**Supplementary Table S1.** Description of Covariates

| Covariates | Description |
| --- | --- |
| Age | In subgroup analysis, age was categorized into two groups:＜60, and ≥60 years |
| Gender | Gender was categorized as male and female |
| Race/Ethnicity | Race was reported as Mexican American, non-Hispanic white, non-Hispanic black, or other race |
| Educational level | Education level was categorized as high school or less, some college, college graduate or higher |
| Marital status | Marital status was reported as married, widowed, divorced, separated, never married, or living with a partner. |
| Family income | Family income was represented by Ratio of Family Income to Poverty, which was divided into three groups: low (≤1.00), medium (1.01-3.00), and high (>3.00). |
| Smoking | Smoking behaviors were based on whether participants had smoked at least 100 cigarettes in their lifetime. |
| Drinking | Alcohol use was defined as consuming at least 12 drinks of any type of alcoholic beverage in any one year. |
| Vigorous activities | The Physical Activity questionnaire recorded whether participants engaged in vigorous recreational activities. Responses were categorized as “yes” or “no”. |
| Energy | Energy intake was determined by averaging 2 days of dietary recall. |
| Hypertension | Hypertension was identified on the basis of Question BPQ020, explicitly asking, “Have you ever been told by a doctor or other health professional that you had hypertension, also called high blood pressure?” |
| Hypercholesterolemia | Hypercholesterolemia was identified on the basis of Question BPQ080，explicitly asking, “Have you ever been told by a doctor or other health professional that your blood cholesterol level was high?” |
| Diabetes | Diabetes was identified on the basis of Question DIQ010, explicitly asking, “Have you ever been told by a doctor or other health professional that you have diabetes or sugar diabetes?” |
| Stroke | Stroke was identified on the basis of Question MCQ160f, explicitly asking, “Have you ever been told by a doctor or other health professional that you have a stroke?” |
| Cardiovascular disease | The cardiovascular disease was obtained from the Medical Conditions questionnaire, which captured if participants had been told by a physician that they had coronary heart disease, congestive heart failure, angia or heart attack |
