# Supplementary material for: Dietary antioxidants and obesity: a new perspective on the role of composite dietary antioxidant index in reducing obesity risk using a dual-criteria definition
Source: Front Nutr. 2025 Jul 31;12:1600925. doi: 10.3389/fnut.2025.1600925 (PMC12350136; doi:10.3389/fnut.2025.1600925)
Supplement: Supplementary file 2 [file Table_2.docx]

**Supplementary Table S2.** Characteristics of Participants in the NHANES 2009-2018 Cycles.

| Characteristic | Participants^a^, No. (%) | | | | | |  |
| --- | --- | --- | --- | --- | --- | --- | --- |
|  | CDAI level | | | | | |  |
|  | Total | Q1  (≤-2.6) | Q2  (-2.7 to -0.6) | Q3  (-0.7 to 1.8) | Q4  (≥1.9) | *P* value | VIF |
| Participants, No. | 17067 | 4239 | 4250 | 4282 | 4296 |  |  |
| Age, mean (SD), y | 48.3 (16.7) | 48.4 (17.3) | 48.8 (16.9) | 48.6 (16.5) | 47.5 (16.3) | 0.083 | 1.874 |
| Gender |  |  |  |  |  | <0.001 | 1.264 |
| Male | 8310 (49.2) | 1433 (32.3) | 1834 (41.7) | 2242 (51.8) | 2801 (66.5) |  |  |
| Female | 8757 (50.8) | 2806 (67.7) | 2416 (58.3) | 2040 (48.2) | 1495 (33.5) |  |  |
| Race/Ethnicity |  |  |  |  |  | <0.001 | 1.202 |
| Mexican American | 2166 (7.8) | 503 (7.8) | 575 (8.3) | 528 (7.1) | 560 (8.2) |  |  |
| Non-Hispanic White | 7447 (68.2) | 1677 (63.0) | 1838 (67.3) | 2019 (71.8) | 1913 (69.3) |  |  |
| Non-Hispanic Black | 3684 (10.5) | 1165 (15.3) | 927 (11.3) | 775 (7.8) | 817 (8.8) |  |  |
| Other Race | 3770 (13.5) | 894 (13.9) | 910 (13.2) | 960 (13.2) | 1006 (13.7) |  |  |
| Educational level |  |  |  |  |  | <0.001 | 1.387 |
| High school or less | 7038 (34.4) | 2214 (47.1) | 1808 (36.3) | 1628 (31.1) | 1388 (26.0) |  |  |
| Some college | 5432 (32.6) | 1358 (34.1) | 1395 (33.9) | 1343 (31.6) | 1336 (31.5) |  |  |
| College graduate or higher | 4597 (33.0) | 667 (18.8) | 1047 (29.8) | 1311 (37.3) | 1572 (42.5) |  |  |
| Marital status |  |  |  |  |  | <0.001 | 1.401 |
| Married/Living with partner | 10338 (64.1) | 2256 (55.4) | 2554 (64.1) | 2768 (68.3) | 2760 (66.8) |  |  |
| Divorced/Widowed/Separated | 3719 (17.9) | 1188 (24.4) | 975 (18.9) | 805 (15.8) | 751 (14.3) |  |  |
| Never married | 3010 (17.9) | 795 (20.2) | 721 (17.1) | 709 (15.9) | 785 (18.9) |  |  |
| Family income |  |  |  |  |  | <0.001 | 1.399 |
| Low | 5034 (20.4) | 1636 (29.6) | 1265 (21.2) | 1112 (17.4) | 1021 (15.5) |  |  |
| Medium | 6466 (34.7) | 1678 (38.7) | 1626 (35.8) | 1628 (33.1) | 1534 (32.3) |  |  |
| High | 5567 (44.9) | 925 (31.7) | 1359 (43.0) | 1542 (49.5) | 1741 (52.1) |  |  |
| Smoking | 7461 (43.1) | 1972 (46.4) | 1782 (42.7) | 1908 (43.9) | 1799 (40.0) | 0.004 | 1.180 |
| Drinking | 13051 (81.5) | 2969 (75.4) | 3196 (80.6) | 3409 (83.3) | 3477 (85.3) | <0.001 | 1.138 |
| Vigorous activities | 3959 (27.7) | 649 (18.4) | 861 (23.9) | 1036 (28.5) | 1413 (37.6) | <0.001 | 1.227 |
| Energy, median(IQR), kcal/d | 1914.5 (1473.5, 2464.6) | 1329.5 (1051.4, 1642.6) | 17830 (1487.3, 2106.5) | 21410 (1787.8, 2525.5) | 2646.5(2105,3235.5) | <0.001 | 1.872 |
| Hypertension | 6530 (33.3) | 1791 (35.8) | 1678 (34.3) | 1553 (32.9) | 1508 (31.0) | 0.037 | 1.340 |
| Hypercholesterolemia | 6420 (35.8) | 1586 (34.8) | 1616 (35.8) | 1673 (36.3) | 1545 (36.0) | 0.827 | 1.246 |
| Diabetes | 2360 (10.3) | 688 (12.1) | 626 (11.1) | 558 (9.8) | 488 (8.8) | 0.016 | 1.151 |
| Stroke | 645 (2.7) | 225 (3.8) | 160 (2.9) | 142 (2.7) | 118 (1.7) | <0.001 | 1.059 |
| CVD | 1458 (6.8) | 442 (7.8) | 370 (6.8) | 349 (6.8) | 297 (5.8) | 0.121 | 1.169 |
| BMI, mean (SD), kg/m2 | 29.3 (6.8) | 29.9 (7.1) | 29.6 (7.1) | 29.3 (6.7) | 28.6 (6.5) | <0.001 | - |
| BMI-Obesity^b^ | 7017 (40.0) | 1917 (44.5) | 1816 (41.8) | 1751 (40.0) | 1533 (34.9) | <0.001 | - |
| WC, mean (SD), cm | 100.2 (16.6) | 100.6 (16.6) | 100.5 (17.0) | 100.4 (16.4) | 99.3 (16.6) | 0.209 | - |
| Abdominal obesity^c^ | 13028 (75.5) | 3278 (76.1) | 3274 (75.7) | 3298 (76.6) | 3178 (73.9) | 0.275 | - |
| BMI-WC-Obesity^d^ | 7002 (39.9) | 1909 (44.3) | 1812 (41.7) | 1749 (40.0) | 1532 (34.9) | <0.001 | - |
| WHtR, mean (SD) | 0.6 (0.1) | 0.6 (0.1) | 0.6 (0.1) | 0.6 (0.1) | 0.6(0.1) | <0.001 | - |

Abbreviations: NHANES, National Health and Nutrition Examination Survey; CDAI, Composite Dietary Antioxidant Index; CVD, Cardiovascular Disease; BMI, Body Mass Index (calculated as weight in kilograms divided by height in meters squared); WC, Waist Circumference; WHtR, Waist-Height Ratio (calculated as waist circumference divided by height).

^a^ Data are presented as unweighted number (weighted percentage) unless otherwise indicated.

^b^ BMI-Obesity was defined as BMI ≥30 kg/m^2^.

^c^ Abdominal obesity was defined as waist circumference ≥88 cm for women or ≥102 cm for men.

^d^ BMI-WC-Obesity was defined as BMI ≥30kg/m^2^ and, female waist circumference ≥88cm or male waist circumference ≥102cm.
