# Supplementary material for: Dietary antioxidants and obesity: a new perspective on the role of composite dietary antioxidant index in reducing obesity risk using a dual-criteria definition
Source: Front Nutr. 2025 Jul 31;12:1600925. doi: 10.3389/fnut.2025.1600925 (PMC12350136; doi:10.3389/fnut.2025.1600925)
Supplement: Supplementary file 3 [file Table_3.docx]

**Supplementary Table S3.** Sensitivity analyses.

|  | Crude |  |  | Model 1^a^ |  |  | Model 2^b^ |  |  | Model 3^c^ |  |
| --- | --- | --- | --- | --- | --- | --- | --- | --- | --- | --- | --- |
|  | OR (95%CI) | *P* value |  | OR (95%CI) | *P* value |  | OR (95%CI) | *P* value |  | OR (95%CI) | *P* value |
| BMI-Obesity |  |  |  |  |  |  |  |  |  |  |  |
| No | Ref | - |  | Ref | - |  | Ref | - |  | Ref | - |
| Yes | 0.96 (0.95, 0.97) | <0.001 |  | 0.97 (0.96, 0.99) | <0.001 |  | 0.97 (0.96, 0.99) | 0.003 |  | 0.97 (0.95, 0.99) | <0.001 |
| Abdominal-Obesity |  |  |  |  |  |  |  |  |  |  |  |
| No | Ref | - |  | Ref | - |  | Ref | - |  | Ref | - |
| Yes | 0.99 (0.97, 1.00) | 0.082 |  | 0.97 (0.95, 0.99) | <0.001 |  | 0.97 (0.95, 0.99) | 0.007 |  | 0.97 (0.95, 0.99) | 0.008 |
| WHtR |  |  |  |  |  |  |  |  |  |  |  |
| ≤0.5 | Ref | - |  | Ref | - |  | Ref | - |  | Ref | - |
| ＞0.5 | 0.94 (0.92, 0.95) | <0.001 |  | 0.94 (0.92, 0.96) | <0.001 |  | 0.95 (0.93, 0.97) | <0.001 |  | 0.94 (0.92, 0.96) | <0.001 |
| Excluding outlier | 0.96 (0.95, 0.98) | <0.001 |  | 0.98 (0.96, 1.00) | 0.053 |  | 0.98 (0.96, 1.00) | 0.053 |  | 0.97 (0.95, 1.00) | 0.023 |

Abbreviations: OR, Odds ratio; CI, Confidence interval; BMI, Body Mass Index; WHtR, Waist-Height Ratio.

^a^ Adjusted for sociodemographic variables (age, sex, race and ethnicity, educational level, family income, and marital status) and NHANES cycles.

^b^ Adjusted for sociodemographic variables, NHANES cycles, and lifestyle variables (smoking status, drinking status, physical activity, and calorie consumption).

^c^ Adjusted for sociodemographic variables, NHANES cycles, lifestyle variables, and comorbidities (hypertension, hypercholesterolemia, diabetes, cardiovascular disease and stroke).
